# Comparison of barriers and facilitators of MIND diet uptake among adults from Northern Ireland and Italy

**DOI:** 10.1186/s12889-021-10307-9

**Published:** 2021-02-02

**Authors:** Deirdre Timlin, Barbara Giannantoni, Jacqueline M. McCormack, Angela Polito, Donatella Ciarapica, Elena Azzini, Melanie Giles, Ellen E. A. Simpson

**Affiliations:** 1 School of Psychology, Ulster University, Ulster, UK; 2 CREA - Council for Agricultural Research and Economics Research Centre for Food and Nutrition, Via Ardeatina, 546, 00178 Rome, Italy; 3 Faculty of Science, Institute of Technology, Sligo, Ireland; 4 Psychology Research Institute, Ulster University, Coleraine, UK

**Keywords:** MIND diet, COM-B model, Dementia, Adoption, Brain health, Behaviour change wheel

## Abstract

**Background:**

The aim of the study was to identify and compare components of the COM-B (capability, opportunity, motivation and behaviour) model, that influences behaviour to modify dietary patterns in 40–55-year olds living in Northern Ireland (NI) and Italy, in order to reduce the risk of cognitive decline in later life.

**Methods:**

This was a qualitative study examining factors influencing Mediterranean-DASH (Dietary Approaches to Stop Hypertension) Intervention for Neurodegenerative Delay (MIND) diet behaviour. This study further elaborated the COM-B components into the 14 domains of the Theoretical Domains Framework to further understand behaviour. Twenty-five Northern Irish and Italian participants were recruited onto the study, to take part in either a focus group or an interview. Participants were both male and female aged between 40 and 55 years.

**Results:**

Thematic analysis revealed that the main barriers to the uptake of the MIND diet were; time, work environment (opportunity), taste preference and convenience (motivation). Culture (motivation), seasonal foods and lack of family support (opportunity) to be a barrier to the Italian sample only. The main facilitators reported were; improved health, memory, planning and organisation (motivation) and access to good quality food (opportunity). Cooking skills, knowledge (capability) and heathy work lunch (opportunity) reported as a facilitator to the Italian sample only.

**Conclusions:**

Cross-cultural differences in relation to psychosocial barriers and facilitators were found in both samples. More barriers than facilitators towards uptake of the MIND diet were found. There is a need for interventions that increase capability, opportunity, and motivation to aid behaviour change. The findings from this study will be used to design a behaviour change intervention using the subsequent steps from the Behaviour Change Wheel.

## Background

The global ageing population is increasing, with approximately 50 million people worldwide currently living with dementia, which is predicted to rise to 131 million by 2050 [1]. The latest statistics on prevalence of dementia in Europe have shown that overall, Italy has the highest percentage (2.09%) of people living with dementia, compared to the average percentage of the rest of Europe (1.55%). In Northern Ireland, approximately 22,000 people are estimated to be living with dementia, which is 1.06% of the population. Longevity is increasing worldwide, therefore, there is an urgent need to identify potential modifiable risk factors such as diet to promote brain health from an earlier age.

There have been several prospective and cross-sectional studies that have attempted to gain insight into the relationship between the Mediterranean diet [2], DASH diet [3] and cognitive function. The Mediterranean diet is characterised by a high intake of plant food (fruit, vegetables, cereals and legumes), olive oil as the main source of fat, a moderate intake of fish, a low to moderate intake of dairy products and alcohol, a low intake of saturated fats, meat and poultry [4]. The DASH diet is similar to the Mediterranean diet, however, compared to the Mediterranean diet, the DASH diet requires high intake of low-fat dairy [5]. Prospective studies in the USA and Europe with both the Mediterranean and DASH diets over several years with older adults found an association with less cognitive decline [6, 7], specifically, improved episodic, semantic, and working memory [8]. Furthermore, several cross-sectional studies in Italy and NI with older adults, found that close adherence to the Mediterranean diet was associated with lower cognitive impairment [9, 10] and better cognitive function [11].

Prospective studies conducted in midlife over an extended 16-year period also showed a significant association with decreased risk of cognitive impairment [12] and improved psychomotor speed over a 4-month period in midlife [13]. Research has found that a healthy diet in midlife is positively associated with cognitive function [14]. Moreover, research on both the DASH and Mediterranean diets have shown promising results in the protection against cardio risk factors for dementia [15]. However, the Mediterranean and DASH diets are not specific to the literature on nutrition and the brain. Therefore, a new diet called the Mediterranean-DASH Intervention for Neurodegenerative Delay (MIND) [16] diet was designed that incorporated many of the basic components of Mediterranean and DASH diet, but with modifications that reflect the best scientific evidence on nutrition and prevention of dementia [17].

The MIND diet promotes 10 healthy foods (Leafy greens, other veg, nuts, berries, fish, poultry, olive oil, beans, whole grains, red wine) and limits 5 other foods (red meat, butter, cheese, pastries and sweets, fried foods). While previous research shows that higher consumption of vegetables are associated with lower risk of cognitive decline [18, 19], the strongest association is observed for higher intake of leafy greens [20, 21]. Previous research on cognitive function or dementia do not observe protective effects for overall fruit consumption [20, 21]. However, berries were shown to slow cognitive decline, particularly in global cognition and verbal memory in older adults [22].

There has been limited research to date investigating the effectiveness of the MIND diet. Morris et al. [23] originally devised the MIND diet and found that the diet can slow cognitive decline over an average of 4.7 years in adults aged 58–98 years old [23]. Interestingly, recent research found that the MIND diet and not the Mediterranean diet, protected against 12-year incidence of mild cognitive impairment and dementia in older adults [24]. Also, a large observational study with older adults found that longer adherence to the MIND diet was associated with better verbal memory [25].

While there is little research on the social, environmental, and cultural perspectives of adopting the MIND diet, social and cultural changes have been shown to have contributed to reversal of dietary habits in Southern European countries, with socio-economic variables highlighted as associated with adherence to a Mediterranean diet [26–28]. Social, cultural, and environmental factors have been found to be barriers in adopting a Mediterranean style diet [29, 30]. British culture has been reported as being non-conducive to a Mediterranean dietary pattern [31], with barriers such as time, work and convenience influencing Mediterranean style diet behaviour [32, 33]. Foods from a healthy dietary pattern may be more expensive to buy than those from a less healthy diet [34, 35], and this may influence people’s food choices [34]. Therefore, a major barrier to consuming a Mediterranean style diet could be budget, especially for those of low socio-economic status. However, previous research has found, that while consuming healthier foods such as increasing wholegrains, fish, fruit and vegetables, may be more expensive, this cost could be reduced by reducing unhealthier foods such as red meat and sugary foods [36]. Identifying barriers and knowledge gaps towards Mediterranean style diet adoption, such as budget, time, convenience, and work, has implications for the design of behaviour change interventions aiming to promote dietary change [29].

As we are looking to promote healthy ageing, we are investigating modifiable risk factors in the prevention of cognitive decline. Research has found that a healthy diet in midlife is positively associated with cognitive function in later years [14, 15]. Therefore, this study could add support to the dementia strategy research by exploring modifiable risk factors in the prevention of dementia, which could be applied globally.

### Theoretical framework

**Fig. 1:**
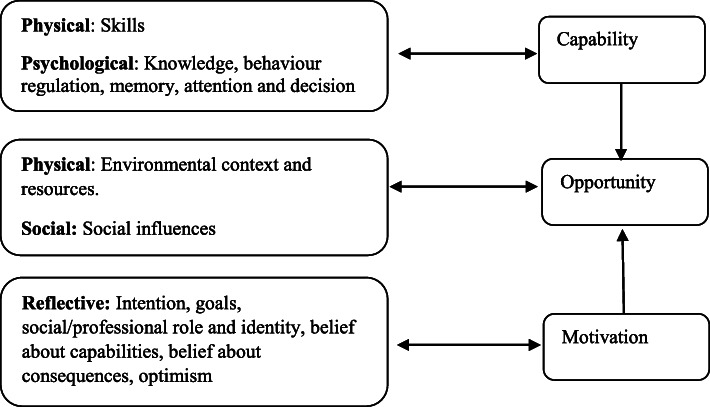
(a): TDF domains and corresponding mapping onto the COM-B component

The theoretical framework underpinning this research is the COM-B model [37]. Changing behaviour involves changing one or more of the components of the COM-B model, which stand for, capability, opportunity, motivation, and behaviour (see Fig. 1). Capability can be either psychological (knowledge, psychological skills, or stamina) to perform the behaviour, or “physical” (having the physical skills, strength or stamina) to perform the behaviour. Opportunity can be divided into “physical” (what the environment allows in terms of time, resources etc) or “social” (interpersonal influences, social cues, cultural norms). Motivation can be divided into “reflective” (self-conscious planning and evaluations, beliefs about what is good or bad) or “automated” (wants and needs, desires, impulse and reflex responses) [37]. The Theoretical Domains Framework (TDF) facilitates understanding of health behaviours around evidence-based guidelines and provides a method to categorise behaviour [38]. Use of the TDF to identify factors influencing MIND diet behaviour can then be mapped onto the COM-B model for designing interventions. The TDF has 14 domains that may influence behaviour change [38] (see Fig. 1).

The COM-B model is at the core of an overarching framework called the Behaviour Change Wheel [37] which is a three-stage approach to designing a behaviour change intervention. This framework includes 9 intervention functions (education, persuasion, incentivisation, coercion, training, restriction, environmental restructuring, modelling, and enablement linked to the COM-B model. These are how an intervention might change behaviour and are linked to behaviour change techniques [37]. BCTs are considered the active component of the intervention designed to change behaviour, such as self-monitoring of behaviour and goal setting. The COM-B model and TDF have been used by several studies to explore barriers and facilitators to behaviour change in sexual health [39], physical activity in obese pregnant women [40] and reducing sugar [41].

Previous research found differences in dietary patterns of people who live in Rome and NI, with NI consuming more ready-made meals [42], snacked between meals more often than Italians [43] and consumed more takeaway food, sugary drinks and less fruits and vegetables that those living in other Mediterranean countries [44]. Although the MIND diet is a hybrid of the Mediterranean and DASH diets, it is a new diet that specifies foods such as berries, leafy greens, and wholegrains, which are not part of a traditional Mediterranean diet. Furthermore, the Mediterranean and DASH diets are not specific to the literature on nutrition and the brain. Therefore, the MIND diet was designed that incorporated many of the basic components of the Mediterranean and DASH diet, but with modifications that reflect the best scientific evidence on nutrition and prevention of dementia [17]. Comparing factors from the COM-B model (capability, opportunity and motivation) that may influence MIND diet behaviour across a Mediterranean and non-Mediterranean country, can reveal valuable insights that highlight diverse habits and beliefs across culture, which may be particularly informative in the development of behaviour change interventions.

The aim of the study was to establish and compare components of the COM-B model that influence the uptake of the MIND diet in a 40–55-year old Italian and Northern Ireland (NI) sample, that will inform a dietary behaviour change intervention.

Specific objectives were:

- To determine participants perceived capability, opportunity, and motivation to the uptake of the MIND diet in 40–55-year olds in a Mediterranean (Italy) and non-Mediterranean (NI) country.
- Compare barriers and facilitators to the MIND diet from a Mediterranean and non-Mediterranean country.
- Identify intervention functions and BCTs that are likely to change MIND diet behaviour.

## Method

### Participants and study design

Part of the methods in this manuscript can also be seen in Timlin et al. [45]. Twenty-five participants from NI (Belfast, Armagh city, County Tyrone) (female 60%, male 40%) [45] and twenty-five participants from Rome, Italy (female 64%, male 36%) aged 40–55 years were recruited onto the study, to take part in either a focus group or an interview. Interview/focus groups took place in person (NI: 15 interviews, 2 focus group *n* = 6, *n* = 4; Italy 13 interviews, 2 focus group *n* = 6 *n* = 6). Ethical approval was obtained from the School of Psychology Staff and Postgraduate Filter Committee at Ulster University, which is in accordance with The Code of Ethics of the World Medical Association (Declaration of Helsinki). Participants were approached by email, Facebook and advertisement booklet, which included some brief information about the study. Interested participants were asked to contact the researcher by email and sent a participant information sheet (PIS), consent form and information booklet on the MIND diet. Questions asked to participants were the same for both NI and Italian populations. Before the Italian interviews began, questions were translated from English to Italian by a fluent Italian speaker (BG). Questions were then back translated to English to ensure the interpretation of questions [46]. Most of the interviews were spoken in English (18) and those that were not were translated during the interviews by one of the Italian researchers (BG), to allow the English-speaking researcher (DT) to transcribe and analyse data from all of the transcripts. All interviews/focus groups were recorded and transcribed verbatim. The oral recordings and transcripts were sent to the Italian speaking researcher to check for missing data. Interviews were conducted in a private room at either a research centre or a community facility such as a library, convenient to the participant. In accordance with the COM-B framework, both focus groups and interviews were conducted [37] using semi-structured questions and lasting between 30 and 60 min each (see Table 1). The interview and focus group questions were based on guidance using the COM-B [37] model and TDF [38] (Table 1). The interview schedule was developed using the COM-B model, and informed the content analyses, as seen in previous research conducted with the NI population of this research, as seen in previous research conducted with the NI population of this research [45]. All participants were asked to complete a personal information form and consent form before the interview/focus group began. The information form contained questions on participants diet at baseline (see Table 2) and showed that those living in NI consumed more red meat, fried food, butter, and sugary foods than those living in Italy. Participants were informed that the study was voluntary and that they could withdraw at any time. They were assured of confidentiality regarding any personal information they supplied to the researcher. It has been suggested by similar theoretical models, that 25 participants is the ideal sample size for qualitative research [47]. Also, similar to other qualitative studies using the COM-B and TDF [39, 40], twenty-five NI and 25 Italian participants were recruited onto the study, to take part in either a focus group or an interview.

**Table 1:** Interview/focus group questions asked to participants in accordance with the TDF and COM-B model

COM-B	TDF	QUESTION
Psychological Capability	Knowledge.	What is your understanding of the MIND diet?
Psychological Capability	Memory, attention and decision processes.	To what extent is eating MIND diet foods something you normally do? ➢ Prompt: Do you eat MIND diet foods each day
Psychological Capability	Behaviour regulation	To what extent do you monitor whether you are eating MIND die foods?
Physical Capability	Skills	To what extent are you confident in cooking/eating MIND diet foods?
Social Opportunity	Social influences	To what extent do/would your family or friends help or hinder you eating MIND diet foods? ➢ Prompt: Does/would your family support you in eating the MIND diet?
Physical Opportunity	Environmental context and resources.	Discuss anything in your work or/and home environment that might help or hinder you eating the MIND diet? E.g budget, time
Reflective Motivation	Social/Professional role and identity	To what extent would eating the MIND diet be accepted by your friends and family? ➢ Prompt: Do you think your family/friends influences what you eat?
Reflective Motivation	Belief about capabilities	How difficult/easy would it be for you to eat the MIND diet? ➢ Prompt: What are the barriers to consuming the MIND diet? ➢ Prompt: What are the facilitators to consuming the MIND diet?
Reflective Motivation	Optimism	To what extent are you confident that any barriers you may have to eating the MIND diet can be solved?
Reflective Motivation	Intention	To what extent do you intend to follow the MIND diet to promote brain health?
Reflective Motivation	Goals	To what extent would you like to follow the MIND diet?
Reflective Motivation	Belief about consequences	What do you think will happen if you eat the MIND diet? ➢ Prompt: Discuss any benefits to eating the MIND diet?
Automatic Motivation	Reinforcement	To what extent are there any incentives for you to the MIND diet?
Automatic Motivation	Emotion	How do you feel about eating the MIND diet?

*COM-B* Capability (C): Psychological or physical ability to enact behaviour, *Opportunity (O)* Physical and social environment that enables behaviour, *Motivation (M)* Reflective or automatic mechanisms that activate or inhibit behaviour, Behaviour (B), *TDF* Theoretical Domains Framework [45].

**Table 2:** Percentage of participants food intake at baseline

	More than once a day		Daily		2–3 times a week		Once a week		Less than once a week	
	Italy	NI	Italy	NI	Italy	NI	Italy	NI	Italy	NI
Fruit & Vegetables %	44	44	26	36	20	20	8	0	4	0
Beans and legumes %	4	0	0	4	44	20	40	24	12	52
Fish %	0	0	0	4	32	28	48	40	20	32
Poultry %	0	4	0	4	36	60	36	34	28	8
Wholegrains %	12	0	16	40	20	16	16	16	36	28
Nuts %	4	0	16	4	12	20	20	32	40	44
Red meat %	0	0	0	8	28	64	40	12	32	16
Cheese %	0	0	12	24	48	48	24	20	8	8
Fried food %	0	0	0	0	4	40	12	24	84	32
Butter %	0	20	0	52	8	12	20	4	72	12
Sweets/pastries %	0	16	8	28	44	20	8	20	40	12

*NI* Northern Ireland N-50, numbers are in percentages.

### Materials and procedure

A topic guide was developed using the TDF. An example of a question related to TDF knowledge was, “what is your understanding of the MIND diet”. A further question exploring participants skills was, “to what extent are you confident in cooking MIND diet foods”. The TDF represents an elaboration of the COM-B’s six components into 14 domains, covering a wide spectrum of behavioural determinants (see Table 1). A booklet containing information on the elements of the MIND diet, and the origins of the diet were given to participants. An in-depth discussion on the MIND diet components was discussed prior to interview and focus groups. All interviews and focus groups were audio recorded.

### Data analyses

The data analyses has been described in full in Timlin et al. [45]. Two researchers (one English speaking and one Italian/English speaking) (DT&BG) independently read through the entire dataset and coded the data from each transcript and assigned initial “code names”. There was a 95% agreement on codes between the two main researcher, which demonstrates an acceptable level of agreement [48]. However, any differences in coding were resolved with discussion between the researchers. Summative content analysis [49] was applied as an additional step in the analysis following agreement of codes. This involved two researchers searching the text for occurrences of codes, that were counted to identify the frequency of each code. Using a common approach [50, 51], TDF domains were judged based on the frequency count of coding for each TDF domain, which had been aggregated from all the factors, beliefs or phrases mentioned that fell within that domain. For example, some participants reported that they believed the MIND diet would make them feel better generally. This belief statement is coded under the TDF domain “belief about consequences.” The frequency coding identified which TDF domains were most commonly reported, establishing the main barriers and facilitators to the uptake of the MIND diet.

## Results

Table 3 reports the characteristics of a total sample, including 25 Italian and 25 NI participants. Transcripts provided data from 12 of the 14 domains of the TDF in the Italian sample, all 14 domains of the TDF in the NI sample and all components of the COM-B model for both samples (see Tables 4 and 5). The most commonly reported barriers and facilitators fell into: Environmental Context and Resources, Belief about Capabilities, Belief about Consequences, Social Influences, Skills and Knowledge. None of the data fell into, reinforcement and goals, which were the least reported domains in the NI study (See Tables 6 and 7 for quotes).

**Table 3:** Summary Characteristics of Interview/Focus Group Participants(*n* = 50)

Characteristic	Northern Ireland (*N* = 25)	Italy (*N* = 25)
Mean age (sd)	44 (4.9)	46 (4.2)
40–44	60 (15)	36 (9)
45–49	16 (4)	44 (11)
50–55	24 (6)	20 (5)
Gender		
Male	40 (10)	36 (9)
Female	60 (15)	64 (16)
Occupation		
Professional	44 (11)	64 (16)
Skilled	16 (4)	36 (9)
Unskilled	40 (10)	0
Education		
Higher education	36 (9)	64 (16)
Further education	28 (7)	36 (9)
No formal qualifications	36 (9)	0
Marital status		
Married	44 (11)	44 (11)
Co-habiting	4 (2)	4 (2)
Separated	4 (2)	4 (2)
Single	32 (8)	32 (8)
Widowed	4 (2)	4 (2)
Children in household		
Yes	44 (11)	72 (18)
No	56 (14)	28 (7)

*Education* Level of education obtained within a discipline or profession. Higher education = undergraduate/postgraduate degree: Further education = any study after secondary school that does not include higher education, such as higher national diploma, higher national certificate, apprentices for industry such as hairdressing, plumbing, *Sd* standard deviation *N* = 50

**Table 4:** Barriers in rank order of utterances in relation to MIND diet in 40–55-year olds in Rome and NI: COM-B and TDF domains

Italy					Northern Ireland				
COM-B	TDF	Rank order	Frequency of Utterances	% of utterances.	COM-B	TDF	Rank order	Frequency of utterances	%of utterances
Physical opportunity	Environmental context and resources	1	93	33	Physical opportunity	Environmental Context and resources	1	90	29
Social opportunity	Social Influence	2	43	15	Reflective motivation	Belief about capabilities	2	46	15
Reflective motivation	Belief about Capabilities	3	37	13	Psychological capability	Knowledge	3	37	12
Psychological capability	Behaviour regulation	4	29	10	Psychological capability	Memory, attention, Decision making	4	30	10
Psychological capability	Knowledge	5	29	10	Psychological capability	Behaviour regulation	5	24	7
Reflective motivation	Social, Professional and Identity	6	15	5	Physical capability	Physical skills	6	17	6
Reflective motivation	Belief about consequences	7	11	4	Social opportunity	Social Influence	7	15	5
Physical capability	Skills	8	9	3	Reflective motivation	Belief about consequences	8	12	4
Reflective motivation	Intention	9	9	3	Reflective motivation	Social professional and identity	9	12	4
Reflective motivation	Optimism	10	7	2	Reflective motivation	Intention	10	9	3
Automatic motivation	Emotion	11	4	2	Reflective motivation	Optimism	11	6	2
Automatic motivation	Reinforcement	0	0	0	Reflective motivation	Goals	12	5	2
Reflective motivation	Goals	0	0	0	Automatic motivation	Emotion	13	3	1
Psychological capability	Memory, attention	0	0	0	Automatic motivation	Reinforcement	14	1	0
			286	100				307	100

Information above the thick black line represents the top 6 reported domains of the TDF and corresponding COM-B components. Eighty percent of the data fell into the top 6 TDF domains, *COM-B* Capability (C): Psychological or physical ability to enact behaviour, *Opportunity (O)* Physical and social environment that enables behaviour. Motivation (M): Reflective or automatic mechanisms that activate or inhibit behaviour; Behaviour (B), *TDF* Theoretical Domains Framework

Utterances: Spoken word/words in relation to themes/subthemes emerging from questions asked regarding MIND diet. *n* = 50

**Table 5:** Facilitators in rank order of utterances in relation to MIND diet in 40–55-year olds in Rome and NI: COM-B and TDF domains

Italy					Northern Ireland				
FACILITATORS COM-B	TDF	Rank order	Frequency of utterances	% utterances	COM-B	TDF	Rank order	Frequency of utterances	% of utterances
Physical opportunity	Environment context	1	48	21	Reflective motivation	Belief about consequences	1	28	17
Reflective motivation	Belief about Capabilities	2	36	16	Reflective motivation	Belief about capabilities	2	27	16
Reflective motivation	Belief about consequences	3	32	14	Physical opportunity	Environmental Context and resources	3	22	13
Social opportunity	Social	4	28	12	Social Opportunity	Social influence	4	21	13
Physical capability	Skills	5	19	8	Physical capability	Skills	5	20	12
Reflective motivation	Identity	6	16	7	Automatic motivation	Emotion	6	15	9
Automatic motivation	Emotion	7	16	7	Automatic motivation	Reinforcement	7	10	6
Reflective motivation	Optimism	8	10	4	Reflective motivation	Intention	8	6	4
Reflective motivation	Intention	9	10	4	Psychological capability	Behaviour regulation	9	4	2
Automatic motivation	Reinforcement	10	7	3	Reflective motivation	Optimism	10	4	2
Psychological capability	Regulation	11	4	2	Reflective motivation	Social/Professional and identity	11	3	2
Psychological capability	Attention	12	3	1	Psychological capability	Knowledge	12	3	2
Psychological capability	Knowledge	13	2	1	Psychological capability	Memory	13	1	1
			231	100				164`	100

Information above the thick black line represents the top 6 reported domains of the TDF and corresponding COM-B components. Eighty percent of the data fell into the top 6 TDF domains; *COM-B* Capability (C): Psychological or physical ability to enact behaviour, *Opportunity (O)* Physical and social environment that enables behaviour, *Motivation (M)* Reflective or automatic mechanisms that activate or inhibit behaviour, Behaviour (B), *TDF* Theoretical Domains Framework

Utterances: Spoken word/words in relation to themes/subthemes emerging from questions asked regarding MIND diet. *n* = 50

**Table 6:** *Quotes from barriers regarding uptake of the MIND diet in rank order*

***Northern Ireland***			***Rome***		
COM-B/TDF	SUB-THEME	QUOTE	COM-B/TDF	Subtheme	QUOTE
Physical opportunity: Environmental context	1. Time 2. Food environment at work/canteen 3. Budget 4. Treats in for kids.	“For me it is time, by the time you get home from work, and maybe have done overtime, you couldn’t be bothered” “There is nothing healthy in a canteen”	Physical opportunity: Environmental context	1. Availability/ Access to food 2. Budget 3. Time 4. Season	“Finding berries and the cost of them are a barrier” “Berries are hard to find as they are seasonal, I only eat them in summer”
Reflective motivation: Belief about capabilities	1. Convenience 2. Taste preference 3. Mindset	“Kids don’t want healthy stuff, so sometimes I have convenience stuff to make it easier for me” “I don’t like fish, you know the strong smelling fishy fish”	Social opportunity: Social influence	1. Family influence 2. Visiting family And friends	“The problem is my family, they only eat white pasta” “I would cook more unhealthily and quantity if family are visiting”
Psychological capability: Knowledge	1. Lack knowledge of MIND diet and foods	“If you don’t know what is healthy for your brain, you won’t eat that way”	Reflective motivation: Belief about capabilities	1. Taste preference 2. Convivence Mindset	I don’t buy the brown pasta as it is more expensive and it doesn’t taste as nice as the white” “I don’t eat vegetables, any kind of them” “I love cheese, I do not think I could eat less cheese”
Psychological capability: Memory, attention and decision process	1. Alcohol 2. Tired 3. Holidays	“If I had a good drink at the weekend, it would take Tuesday or Wednesday to get over it, and I wouldn’t want to eat this food”	Psychological capability: Behaviour regulation	1. Self-monitoring	“No, I don’t monitor my food intake”
Psychological capability: Behaviour regulation	1. Lack monitoring of food consumption	“No, I don’t, and sure, when I go to weight watchers, I don’t even do it”	Psychological capability: Knowledge	1. Lack knowledge of MIND diet.	“I have never heard of the MIND diet”
Physical capability: Skills	1. Lack cooking skills	“I couldn’t cook that, if you handed me all the ingredients, I would be like, what am I doing with it”	Social, professional and identity.	1. Culture	“My family eat lots of food, lots of white pasta and cheese, this is typical of Southern Italians to eat more and are more overweight” “Berries are not part of our culture”

*COM-B* Capability, Opportunity, Motivation, Behaviour, *TDF* Theoretical domains framework

**Table 7:** *Quotes from participants regarding facilitators of uptake of the MIND diet*

***Northern Ireland***			***Rome***		
COM-B/TDF	SUBTHEME	QUOTE	COM-B/TDF	SUBTHEME	QUOTE
Reflective motivation: Belief about consequences	1. Feel better generally 2. Improve psychological health 3. Improve memory	“I think the diet would just help you feel better generally” “And even help your head, less stress and worry”	Physical Opportunity: Environmental context and Resources	1. Bring lunch 2. Time	“Here I bring lunch every day, it is very simple for me to prepare my salads so not a barrier” “Having the time to travel to get better quality food would be a facilitator”.
Reflective motivation: Belief about capabilities	1. Planning/ preparation/ organisation	“Organisation and preparation the night before, so having your berries and salad ready for work”	Reflective motivation: Belief about capabilities	1. Normal diet 2. Simple meals 3. Organisation 4. Motivation	“sometimes it is easier for all the family if you can cook it quickly, like pasta and veg” “If you were motivated enough, I think you could overcome your barriers”. “I think you need to plan and be motivated”.
Physical opportunity: Environmental context and resources	1. Accessibility fresh/frozen food 2. Bring lunch to work	“I would go to Lidl, because it is cheaper and better quality” “In my work, you need to be prepared and bring lunch with you”	Reflective motivation: Belief about consequences	1. Overall health 2. Cholesterol 3. Lose weight 4. Fiber/bowel	“I think this diet could help you gain more health” “I think my bowels would work better on this diet” “I think with eating less cheese would be good for your cholesterol” “I think you could lose weight on this diet”
Social opportunity: Social influence	1. Family support/influence	“My mum is always cutting out articles showing me research on good and bad foods for your health.	Social opportunity: Social influence	1. Family support/ influence	“Yes, my wife would support me if I wanted to do this diet” “yes, I think if I was out with family, there would be more alcohol, unhealthy foods and less veg”
Physical capability: Skills	1. Confident cook	“I am pretty confident cooking these foods”	Physical capability: Skills	1. Confident cook	“Yes, I cook generally the same legumes, I don’t like beans very much so I don’t cook them often, but I am able to cook them”
Automatic motivation: Emotion	1. Positive	“I would be positive about it, I get excited trying new things”	Reflective motivation Professional, social and identity	1. Culture	“this is typical foods for me, this would not be difficult for me” “we don’t eat butter, it is not in our culture, we use olive oil”
			Automatic motivation Emotion	1. Positive	“I would feel positive about doing this diet”

*COM-B* Capability, Opportunity, Motivation, Behaviour, *TDF* Theoretical domains framework

### Capability

Psychological capability was a COM-B component identified as a barrier to adherence to the MIND diet. Twenty percent of the barriers in the Italian sample fell into the psychological component of the COM-B model compared to 29% in the NI sample. These barriers fell into 2 of the TDF domains, behaviour regulation and knowledge. None of the Italian barriers fell into attention and decision process domain, unlike the NI sample, where 10% of barriers fell into this domain.

#### Knowledge

Similar to the NI sample, all Italian participants reported never having previously heard of the MIND diet. Italian participants reported that they recognised that the MIND diet was similar to the Mediterranean diet and to their own diet.

#### Behaviour regulation

This domain is defined as ***“****anything aimed at managing or changing objectively observed or measured actions”* [38], such as self-monitoring. In both samples, most of the participants did not monitor their food intake. However, some participants reported that they use to record their food intake to monitor what and how much they ate but are now able to control their diet from memory.

#### Physical capability: skills

Physical skills are defined as the level of self-efficacy in cooking/eating with MIND diet foods. Skills were reported as a facilitator in both the NI (12%) and Rome samples (8%). Skills were reported as a key barrier only in the NI sample, with 6% of barriers falling into this domain. All participants in the Rome sample reported being confident cooks, even if they didn’t like or cook certain foods, whereas, in the NI sample, it was reported that those who couldn’t cook generally were married men and those participants who reported that they didn’t like certain foods, were not confident in cooking them.

### Opportunity

According to the COM-B model, for behaviour to occur, there must be a physical and social opportunity in the environment. Barriers relating to physical opportunity were the most commonly reported barriers in both the NI and Italian populations, with 29% of all utterances falling into this component in the NI sample and 33% in the Italian sample. The TDF domain related to this component is; environmental context and resources. Social opportunity was reported as being a key barrier and facilitator in both the NI and Italian samples, with 13% of all facilitators and 5% of barriers falling into this component from the NI sample, 15% of all barriers and 12% of facilitators from the Italian sample. The TDF domain related to this component is social influence.

#### Environmental context and resources

This domain is defined as any circumstance of a person’s physical environment or situation that could support or hinder the development of skills and abilities [38]. For example, budget, time, inability to cook or shop, availability of quality foods. The work environment was reported as a barrier to eating the MIND diet foods by both NI and Italian samples. It was reported that canteen food can be unhealthy and that there is the temptation to eat more quantity of food. Several participants reported that if they did not have lunch with them, they would eat out in a café or buy lunch from a bakery which would less healthy. Time was a major barrier reported by both samples, particularly for those that were in employment, however, their reasons for *time* being a barrier differed. For the NI participants, it was more a matter of convenience that they had been working all day, having maybe taken children to after school activities, and did not have the *time* to cook with fresh foods. The Italian population reported *time* as barrier in the same manner, but also, the *time* to travel to access fresh food in the farmers markets in the country, especially for those living in the city.

Budget was also reported as a major barrier to buying several of the MIND diet foods such as fish, berries, and nuts in both populations. However, this was only the view of those participants in low paid jobs or unemployed in the NI sample. Several participants from the Italian sample, who were all professional or skilled workers, reported *budget* to be a barrier, especially with regards to fish and wholegrains.

Treats such as cakes and sweets in the home and workplace were reported as being a major barrier in adhering to the MIND diet in the NI sample. Participants reported that having *treats* in the house for guests and children would hinder them in adhering to the MIND diet as they often eat the *treats* themselves. Also, NI participants reported that *treats* in the workplace were common, that there were always biscuits available and that this would be a hindrance to adhering to the MIND diet. However, treats in the workplace were not reported by the Italian sample, in fact, when asked if biscuits were commonly found in the workplace, participants reported that it was only on occasion that biscuits or treats were offered at work, such as, someone’s birthday.

A major barrier reported by the Italian sample and a key difference between both samples, was access and availability of certain foods of the MIND diet. Most Italian participants reported that the availability of berries out of season were scarce. One participant reported that, Italy provides so many different, tasty fruit, that they would not choose berries that were hard to find and expensive. Several participants also reported that wholegrains were expensive and hard to find. Italian participants also reported that access to fresh fruit, vegetables and fish may hinder them in adhering to the MIND diet, especially those that lived in the city of Rome. Participants reported that the fish and fruit produce in the city is more expensive and poorer quality than in the country and that they would consume less of these because of this reason. In contrast, the NI sample reported that the fruit and vegetables were more expensive and of poorer quality in the country and small towns, and that they would have to travel to the bigger stores to access cheaper better-quality food.

Both samples reported that bringing their lunch to work, would help facilitate adherence to the MIND diet. Participants reported, that in order to consume the MIND diet at work, they would need to bring their own lunch to prevent them from eating out. Many participants from the Italian sample already brought a healthy lunch to work, such as salad, which they perceived would help prevent barriers in adhering to the MIND diet, as they could take a lunch to work containing MIND diet foods.

#### Social influence

This domain is described as the *“interpersonal processes that can cause individual to change their thoughts, feelings or behaviours, which may be due to social pressure, norms, social/family support or peer pressure”* [38]**.** A key barrier reported by both samples was visiting family/friends. Both samples reported that either going out to visit friends or family coming to visit resulted in eating unhealthier and more quantity. However, the NI sample reported eating more fast foods, while the Italian sample reported cooking more unhealthily, such as lasagne, cheese and pasties and more quantity. Family support/influence was reported as a key facilitator by both samples. Participants from NI sample reported that they felt their family would support them if they were to uptake the MIND diet. Another key barrier under this domain which was only reported by the Italian sample, was *lack of family support/influence*. Participants often reported avoiding certain foods such as wholegrains or eating less healthy foods such as vegetables, as other family members did not like them. Also, several participants reported that their family would not support them in this diet, particularly those who originate from the South of Italy, where eating more food and more unhealthily is typical of their culture.

### Motivation

Motivation is a component of the COM-B model and there must be strong motivation for the behaviour to occur [33]. Participants reported reflective motivation to be a barrier to the uptake of the MIND diet and 18% of barriers fell into this component of the COM-B model, compared to 15% in the NI study. More facilitators were reported under this domain with 33% from the NI sample and 37% from the Rome sample.

#### Belief about capabilities

The extent to which the individual believes they were able to adhere to the MIND diet. Taste preference was reported as a major barrier to the adherence of the MIND diet in both the NI and Italian populations. Participants reported not liking various elements of the diet such as fish, vegetables, and chicken. However, many of the participants in the Italian sample reported not liking wholegrains, in particular, wholegrain pasta or bread and even if they did like it, they would not buy it as their children did not eat it. Convenience was also reported as a barrier to the uptake of the MIND diet in both samples. Both samples reported cooking less healthy food to suit their children and eating it themselves rather than making two meals for *convenience*.

Mindset was reported by both samples as a barrier to the uptake of the MIND diet. The NI sample reported that being in the right mindset was important to change diet and to be determined to do so. However, the Italian sample reported the difficulty they perceived in reducing certain foods, such as cheese. Many Italian participants reported that they would not be able to do this. Belief about capabilities were also reported as being a major facilitator in the uptake of the MIND diet with 16% of all barriers falling into this domain in both samples. While both samples reported that being organised and prepared when cooking meals or having lunch prepared for work was a facilitator, the Italian participants reported that the MIND diet seemed similar to their own diet and would be easy to follow. They also reported that the MIND diet allowed for simple meals such as pasta and vegetables which is quick and easy to make.

#### Professional, social and identity

How the individual viewed the uptake/maintenance of the MIND diet relative to their identity (for example, parent, culture). Culture was reported as both a barrier (3%) and a facilitator (7%) under this domain from the Italian sample only. Participants reported that the MIND diet was similar to their own diet and the Mediterranean diet. Participants reported that as they ate most of these foods, that this would help them adhere to the MIND diet. They also reported that butter is not part of their diet, they only use olive oil which further supports uptake of the MIND diet. However, most participants reported that not only were berries hard to find out of season, but they were not part of their culture. Some participants also reported that wholegrains were not part of their culture and it would not be acceptable to serve wholegrains to family and friends. It was also reported that cheese is a big part of the Italian culture and reducing cheese would be difficult to do.

#### Belief about consequences

This domain is described as, anticipated outcomes of not eating brain healthy foods, anticipated or experienced outcomes of eating brain healthy foods. (positive or negative). Belief about consequences was reported as a major facilitator in both samples with it being the most reported facilitator in the NI sample (17%). Both samples reported that if they adhered to the MIND diet, they believed it would be good for their overall health, less sleepy and improve mental health. However, some of the Italian participants recognised that with more fibre from the wholegrains and less cheese, that this would have a benefit for their bowels and cholesterol.

#### Emotion

Both samples reported that they would feel positive about following the MIND diet with 7% of facilitators falling into this domain in the Italian sample and 9% in the NI sample. However, similar to NI participants, even though participants felt positive about the MIND diet, this did not necessarily coincide with their intention to uptake the diet.

## Discussion

To our knowledge, this is the first study investigating adherence to the MIND diet at midlife (40–55 years old) in a Mediterranean and non-Mediterranean country. This study addresses this gap in the literature and highlights cross-cultural perceived barriers and facilitators to adhering to the MIND diet at midlife. Results found that the main barriers and facilitators reported were; environmental context and resources, belief about capabilities, social influence, behaviour regulation, knowledge, skills, belief about consequences, emotion, memory, attention and decision making, and professional, social identity, which can be mapped onto the COM-B model (see Fig. 1). This is the first study to use the COM-B model to code and analyse cross-cultural qualitative responses from individuals at midlife regarding MIND diet behaviour. The reason for this, was to ensure our findings were grounded in theory and identify the main components of an intervention that could change and maintain behaviour.

Similar to the NI sample, the Italian key barriers reported were: environmental context and resources, belief about capabilities, behaviour regulation and knowledge. However, skills, and memory, attention and decision processes were not reported as key barriers in the Rome population. Instead, social influence and social, professional and identity were reported as key barriers to the uptake of the MIND diet. Key facilitators reported were environmental context and resources, belief about capabilities, belief about consequences, social influences, skills, and emotion. The Italian sample reported one further facilitator which was social, professional and identity. Our results confirmed previous research finding regarding commonly reported barriers and facilitators to adherence to healthy dietary change, including *budg*et [52], *time* and *taste preference* [53] and *family influence* [54].

Similar to the NI population, the Italian sample reported having no knowledge of the MIND diet prior to the study or what constituted brain healthy food. Nicklas et al. [55] found that lack of knowledge regarding dietary recommendations and health benefits were reported as a key barrier in meeting dietary recommendations, and lack of information on healthy food was also reported as a major barrier [56].

Lack of monitoring food intake was reported by both samples, highlighting “capability” as major barrier to the uptake of the MIND diet. Previous research found an association between behaviour regulation and changes in dietary outcomes [57], with self-monitoring specifically associated with a positive change in diet [58, 59]. Self-monitoring is shown to not only increase awareness of eating patterns [60, 61], but also allows professionals to identify food aversions/intolerances and poor food choices [61].

Opportunity was highlighted as a key barrier to the uptake of the MIND diet. The main difference between the two samples was due to social influences being reported as a barrier in the Italian sample but not the NI sample. Environmental context and resource was a major theme to emerge with “Time” being a key factor in both samples, mainly reported by those who led busy lives. This finding supports previous research that found “Time” to be a barrier to eating a healthy diet [62, 63]. Busy lifestyle was found to be associated with less home cooked meals [56] and poorer eating habits [64–66].

“Budget” was also found to be a significant barrier in both samples, which was mainly due to the healthy elements of the MIND diet, such as fish, wholegrains, berries, and nuts. These findings support previous research that found the cost of food to be a significant factor in people’s choice of food and consumption [34], and that higher adherence to a whole dietary pattern such as the Mediterranean diet, had higher cost associated with the healthy elements of the diet (fish, fruit, vegetables, nuts), and lower cost to the unhealthy elements of the diet (processed meat and sweet) [56, 67]. These findings are further supported in Roa et al. [35] that found unhealthy processed foods to be less expensive than fruit, vegetables, and nuts. However, Roa et al. [35] explained that the higher cost could be offset by reducing the amount of unhealthy food consumption. Further support for this was found in Germani et al. [68] who compared the cost of a 4-member family with the cost of the same family following the Mediterranean diet and found that the cost of the Mediterranean diet was slightly higher in the overall budget. However, following an increase in the budget for healthy foods such as fruit and vegetables and reducing the budget for unhealthy foods such as pastries and processed food, the overall cost for both diets were similar. It was therefore concluded that low adherence to the Mediterranean diet was not associated with cost but a difference in allocating money to different food groups.

Access and availability of fresh food was reported as both a barrier and facilitator in both samples. However, the Italian sample reported it as a major barrier compared to the NI sample and for different reasons, mainly due to seasonal foods being unavailable and limited access to fresh foods reported by those living in the city. One interesting difference between the two samples under this barrier is that in NI, there is cheaper, better quality food in the bigger stores and cities. However, it was reported that it is in the country markets in Rome that cheaper, fresher food is found. The literature generally supports that access to fresh cheaper foods are a barrier in rural areas. Previous research found that shops selling healthier food was a long distance from country communities [69, 70], and that limited access to food resources led to poorer dietary habits [71].

However, in line with our findings with the Italian sample, previous research found that those who had access to farmers markets or grew their own food, was a facilitator to healthy eating [72]. However, the Italian sample further reported that farmers markets only open in the morning which did not suit those who worked. This finding is supported in Smith et al. [73], that found farmers markets to have inconvenient times and low frequency. Barnridge et al. [74] found that participants reported eating the recommended daily fruit and vegetables when receiving nutrition education and access to a garden. However, those who received no nutrition education but access to the garden, did not report eating the recommended fruit and vegetable, suggesting that it is knowledge not access to the garden that was related to an increase in fruit and vegetable consumption.

Social influence was reported as a barrier to the uptake of the MIND diet by the Italian sample only, and as a facilitator by both samples. Family influence was reported as key barrier in the Italian sample. This may be due to the Italian sample being influenced by their children with 72% of the sample having children in the home compared to only 44% of the NI sample. The Italian sample often reported that their children would not eat certain elements of the MIND diet such as wholegrains or vegetables, influencing their decision to buy or cook such foods. This finding is supported in the literature that the taste preference of family and friends is a barrier to healthy eating [56]. Furthermore, research found the preference of children and family to be an important barrier when adopting a healthier lifestyle, particularly with increasing consumption of healthy foods. However, family support and influence were also reported as a key facilitator in adhering to the MIND diet, which is consistent with previous research that found family support was associated with healthier foods [75, 76].

Motivation was highlighted as a barrier and facilitator to the uptake of the MIND diet in both samples. A major barrier reported in both samples was belief about capabilities, with taste preference being a factor associated with adhering to the MIND diet. This finding is in line with previous research that found taste preference to be a barrier to healthy eating [56]. Morrow et al. [77] found that men were more likely to eat healthily if they did not perceive taste to be a barrier. Many of the Italian participants reported that the MIND diet was very similar to their own diet and therefore, felt it would be quite easy to follow. Previous research found that level of education is associated with healthy eating [78–80] and the Italian sample are all educated with 76% of the Italian sample with a higher qualification compared to 36% of the NI sample with a higher qualification and 36% with no formal qualifications. Research found that level of nutritional knowledge is associated with length of education and awareness of food related issues, leading individuals to be more interested in a balanced dietary pattern [27, 28]. However, the Italian sample perception of the MIND diet adherence ease may be attributable to their culture. The MIND diet is a Mediterranean style diet and many of the Italian participants reported following their cultural diet which is rich in fruit, vegetables, nuts, grains, and olive oil, and that this in itself is a facilitator to adhering to the MIND diet. Research in the Mediterranean countries have found that the Mediterranean diet is progressively disappearing [4, 81]. However, research estimating adherence to the Mediterranean diet in the Mediterranean countries using secondary data, found that Italy had the best adherence to the Mediterranean diet [82]. Even though Italians had the best adherence to the Mediterranean diet, it was still decreasing since the economic crisis [83].

Culture was also reported as a barrier to adhering to the MIND diet in the Italian sample only. Participants often reported that certain MIND diet foods were not typical of their culture and serving certain foods to family and friends were not acceptable, such as wholegrain pasta and bread. This finding is in support of previous research that found low consumption of wholegrains in a Spanish sample [84, 85]. Baruth et al. [86], found family to be a barrier to healthy eating. It was reported in Baruth’s study that pressure to eat more, and the expectation that women would not lose their curves, were barriers to healthy eating. Furthermore, the sample in Baruth’s study was with African American women, and as food is a big part of socialising, and eating traditional food is an important to their cultural identity, African American women may feel pressure to eat more [86].

The findings from this study are important to understand behaviour in the context in which it occurs. These findings not only highlight the components of the COM-B/TDF that need to change in order change behaviour, but the cultural differences in terms of important factors that need addressed in intervention design. The development of an appropriate intervention depends on the understanding of MIND diet behaviour in context, and the findings from this study provides us with the necessary knowledge of factors influencing behaviour that will inform an intervention. This is important, as an intervention to change MIND diet behaviour in Northern Ireland, may not address the needs of those living in Italy. The COM-B model is at the core of an overarching framework called the Behaviour Change Wheel [37] which is a 3-stage systematic approach to designing a behaviour change intervention. The research in this paper represents stage one, to understand behaviour in the context in which it occurs and identify what needs to change in order to change MIND diet behaviour.

Stage 2 identifies the best intervention functions that are most likely to be effective in changing the target behaviour in context. We found that 5 of the 9 intervention functions suggested by the BCW were most relevant to the COM-B behavioural analysis conducted in this study. The 5 intervention functions were: education (increasing knowledge), training (imparting skills), persuasion (influencing attitudes and actions), enablement (providing support to overcome barriers) and environmental restructure (to provide cues and prompts for desired behaviour) [37].

The third stage helps identify content of the intervention by selecting the most appropriate behaviour change techniques which best serve the intervention function. The Behaviour Change Technique Taxonomy v1(BCTTv1) [87], and the theory and techniques tool [88], identified which BCT’s have direct links to the TDF domains being addressed in the MIND diet intervention. For example, the tool showed that there was a link between TDF behaviour regulation and self-monitoring of behaviour. Fifteen BCT’s were identified as likely to be effective in delivering the intervention functions and bringing about change in MIND diet behaviour. Therefore, capability to promote adoption of the MIND diet will be addressed by offering demonstration and instruction on how to perform the behaviour, such as recipes, information on MIND diet food frequency and portion sizes. Opportunity to promote adoption of the MIND diet will be addressed by adding objects to the environment, prompts/cues, remove aversive stimuli such as removing unhealthy snacks, bringing lunch to work and social support. Motivation to promote adoption of the MIND diet will be addressed by a range of self-regulatory BCTs such as goal setting, problem solving, self-monitoring, action planning and information on health consequences. In particular, self-monitoring resources to enable individuals to track their MIND diet behaviour and setting particular goals to meet the weekly MIND diet guidelines.

### Strengths

To our knowledge, this is the first study to develop a “behavioural diagnosis” of factors influencing the uptake of the MIND diet in a Mediterranean and non-Mediterranean country. This was the first study to apply the TDF to explore people’s attitudes towards a whole dietary pattern and compare these attitudes between a Mediterranean and non-Mediterranean country. The COM-B model provides a more comprehensive explanation of adherence than existing models [37], making it easier to identify appropriate interventions. The COM-B model was used as an additional step in the data analysis, increasing the efficiency of the study and showing the framework to be adequate for its purpose.

### Limitations

This study was undertaken in a small sample of Italian and Northern Irish men and women. Our findings in terms of barriers and facilitators reported are “perceived” and context based. Therefore, not only may the findings have limited value in predicting MIND diet behaviour, but also not be generalisable to the whole populations. However, generalisability was not the main aim of our study, rather to explore people’s attitudes and perceptions towards the uptake and adherence to the MIND diet, with the aim to inform an intervention. Another limitation of the study may be researcher subjectivity; however, two researchers identified the codes from the data, suggesting that the themes drawn have credence beyond the lead researcher’s interpretation. Focus groups run the risk of introducing bias [89], resulting from an individual’s desire to conform to social acceptability [90]. However, the focus group participants in this study were acquaintances, reducing the risk of social desirability. A limitation of this study is that the two samples differ in terms of socio-economic status, with all the participants from the Italian sample being of high socio-economic status and approximately one-third of the NI participants of low socio-economic status, which may make comparisons more difficult. Further research should include participants across different socioeconomic backgrounds. Furthermore, half of the Italian participants spoke in Italian and some of the richness of the data may have been lost in translation. However, the second researcher (Italian) translated, transcribed, and analysed the data to maximise interpretation and understanding of the data.

## Conclusion

The COM-B and TDF makes a novel application to understanding what would influence the uptake of the MIND diet. This research identified that the main barriers to the uptake of the MIND diet were; time, work environment (opportunity), taste preference and convenience (motivation), with culture (motivation), seasonal foods and lack of family support (opportunity) to be a barrier to the Italian sample only. The main facilitators reported were; improved health, memory, planning and organisation (motivation) and access to good quality food (opportunity). Cooking skills, knowledge (capability) and heathy work lunch (opportunity) being a facilitator to the Italian sample only. Developing interventions that target these salient barriers to MIND diet uptake will have greater potential to change behaviour. Following detailed behavioural analysis, we used the subsequent stages of the Behaviour Change Wheel to identify 5 intervention functions and 15 BCTs to address the barriers and facilitators to the uptake of the MIND diet.

The findings from this study recommends providing behaviour regulation techniques, such as self-monitoring of MIND diet behaviour to keep track of adherence to MIND diet recommendations, education to increase knowledge of MIND diet and its components, improve skills by providing recipes and weekly food planner, and advice on how to include family in the promotion of MIND diet behaviour. Further strategies to overcome barriers to MIND diet behaviour are to provide advice on planning meals ahead of time to encourage adherence to the MIND diet, provide information on how to overcome workplace diet traps, such as bringing lunch to work and removing unhealthy snacks from work-desk. Future research can use the insight from this paper to test the effectiveness of the intervention functions and BCTs outlined in these findings. Furthermore, understanding barriers and facilitators towards uptake of the MIND diet may help health professionals working with individuals/communities to help prevent or reduce the risk of cognitive decline.

## Abbreviations

AD: Alzheimer’s Disease
BCW: Behaviour Change Wheel
COM-B: Capability, Opportunity, Motivation and Behaviour
DASH diet: Dietary Approaches to Stop Hypertension
HAPA: Health Action Process Approach
HBM: Health Belief Model
Med diet: Mediterranean Diet
MIND diet: Mediterranean-DASH Intervention for Neurodegenerative Delay
NI: Northern Ireland
PIS: Participant Information Sheet
SCT: Social Cognitive Theory
SDT: Self-Determination Theory
TCS: Theory Coding Scheme
TDF: Theoretical Domains Framework
TTM: Transtheoretical Model
TPB: Theory of Planned Behaviour
UK: United Kingdom
USA: United States of America
